# Categorization-driven perceptual learning changes perceived similarity in the naturalistic domain of skin lesion diagnosis

**DOI:** 10.1038/s41598-026-59055-z

**Published:** 2026-07-31

**Authors:** Victoria L. Jacoby, Philip J. Kellman

**Affiliations:** 1 Department of Psychology, University of California, Los Angeles, Los Angeles, USA; 2 Department of Surgery, David Geffen School of Medicine, University of California, Los Angeles, Los Angeles, USA

**Keywords:** Categorization, Perceptual change, Perceptual learning, Representational change, Neuroscience, Psychology, Psychology

## Abstract

Prior work suggests that category learning can reshape perception by shifting perceived similarity among instances. Most evidence comes from artificial stimuli and small category sets. We examined perceptual change in a complex, real-world task: classifying benign and cancerous skin lesions. Medically naive participants rated pairwise similarity among instances from 10 categories before and after comparison-based training with > 500 examples. Training increased perceived similarity for same-category pairs (within-category compression) and decreased it for different-category pairs (between-category expansion). The magnitude of perceptual change predicted generalization to novel instances: greater compression was associated with higher post-training accuracy, and greater expansion with lower false-alarm rates. A label-overlap manipulation showed no differential change, arguing against strategic label retrieval. These findings indicate that perceptual learning systematically reshapes category representations and perceptual experience—promoting within-category compression and between-category expansion—in a challenging, naturally structured domain.

**Supplementary Information:**

The online version contains supplementary material available at https://doi.org/10.1038/s41598-026-59055-z.

## Introduction

Perception is a dynamic process; it changes to discover and prioritize information. Often this involves categorization: Under the guidance of task and environmental demands, perceptual learning processes identify and come to preferentially extract features that group and separate objects in ways that correspond to meaningful structural kinds in the world. New perceptually based categories may be formed, and instances of categories whose basis extends beyond perception are more accurately and efficiently assigned. Categorization is crucial for organizing the vast amounts of information encountered daily, guiding our interactions, and enabling more effective decision-making.

Considerable research has demonstrated relationships between visual perception and categorization (e.g.,^1–7^). Claims about the nature of the relationships have varied. Many experimental results have been interpreted as showing that perception changes to optimize the classification of stimuli (e.g.,^1,2,8,9^). Category learning affects the ability to discriminate between graded stimulus values, with observers typically showing enhanced discrimination at learned category boundaries—a pattern commonly known as *categorical perception*^6,10–14^(for a review, see^15)^. Additional research has reported neural adaptations consistent with these changes^16–18^.

Other work has sought to directly characterize changes in *perceptual experience* that accompany perceptual learning effects^1,3,6,19^. These studies have produced evidence that perceived similarity of instances within and between categories is altered through categorization training. Two characteristic outcomes are *within-category compression*, wherein items belonging to the same category are perceived as increasingly similar after categorization training, and *between-category expansion*, wherein items from different categories are perceived as more distinct^3,11^. These changes have been described in the literature as *perceptual change* and alternatively as *representational change*. We see these as going hand in hand, assuming that perceptual experience is based on encoded descriptions or representations resulting from perceptual processes. For simplicity, we will primarily refer to the relevant phenomena as “perceptual change.”

What drives these changes in perception? A longstanding view is that processes of perceptual learning progressively discover and select information that is most consequential for important groupings and distinctions^8,20,21^. These ideas have been implemented in successful models of perceptual learning that work via selective reweighting of inputs in a neural network^9^. The same general approach characterizes deep convolutional neural networks, which have achieved remarkable success in object classification tasks^22^. It also has much in common with the view that categorization shapes perceptual or psychological space by shifting attention toward properties most relevant for distinguishing category members (e.g.,^23–25^).

A note on terminology: Some researchers on perceptual learning in basic visual tasks have considered only the initial activations in the earliest cortical areas to be the “encoding” or “representation” of the input. In vision, this most often refers to activity in neural units sensitive to oriented contrast in visual area V1. If V1 activations are the sole “representation” of the stimulus, then only modifications of V1 receptive fields can comprise a representational or encoding change. If this is postulated, then all learning effects beyond receptive field modification are held to be decisional, not perceptual, in nature. (We thank one of the anonymous reviewers for noting that this view remains common in computational neuroscience.) There are important conceptual issues here, in part because a preponderance of research findings in visual perceptual learning is more consistent with differentiation^8^ or selective reweighting accounts (e.g.,^9^) than with changes to V1 receptive fields (for a review, see^21)^. In what follows, we interpret our results in ways that are also consistent with selective reweighting accounts of perceptual learning. Crucially, we do not restrict encoding or representation to the earliest sensory activations. Perceptual change may involve perceptual representations at any level of the visual hierarchy that supports perceptual appearance and perceived similarity. We digress briefly to explain why these views are plausible.

The idea that such changes are perceptual, not decisional, is consistent with a large body of work in vision science in which encoding and representation explicitly refer to intermediate- and higher-level perceptual structures, including contours, shape primitives, spatial layouts, events, and object-centered representations (e.g.,^26–28)^. For example, Marr’s^26^ approach has levels of primal sketch, 2.5D sketch, and 3-D model that are fundamentally representational, but importantly, V1-like feature maps are *not* representations that Marr treats as perceptually meaningful; they are *inputs* to representations. Likewise, in Treisman’s feature integration theory, features are not percepts; objects and bound representations are^29^. In the domain of visual perceptual learning, Garrigan & Kellman^30^ showed that learning depends on constancy-based representations (e.g., the perceived size of an object as opposed to its retinal size) and does not appear possible based on early activations prior to constancy. Perceptual constancy is typically the basis for our perceptual experience (e.g., we experience perceived size, lightness, and motion of objects, rather than their retinal size, retinal luminance, or retinal displacement), and these constancies are computed and represented beyond V1. Recent work in shape perception implicates abstract representations as what gets encoded and represented, whereas the sensory activations over which abstract representations are computed are not retained in any durable form^31^. Most work in perceptual organization treats border ownership, interpolation, grouping, and object formation as involving explicit perceptual representations, not decision-stage biases. Conversely, there is substantial doubt that initial activations, which are transient and fluctuate several times a second with eye movements, constitute durable encoding or representations at all^31–34^.

In what follows, we consider evidence for changes in perceived similarity, and we view such changes as depending on alterations to higher-level perceptual representations rather than merely shifting decisional criteria. Perceptual learning alters higher-level perceptual representations, as it changes the inputs from which they are constructed. As far as we know, the only objection to such an arrangement is a belief that some may hold that nothing beyond initial cortical (V1) activations in vision counts as a representation, a view that we believe is palpably false. Ransom^35^ has recently argued this point persuasively in the domain of perceptual learning. As we will see, this view is also highly consistent with our data and with earlier work on this topic^1,3^. Besides interpreting their data in way that is consistent with our view of representations, Ashby, et al.^1^ also reviewed neuroimaging work (e.g^17,36^, that indicates that category learning produces “increased discriminability of their [categories’] neural representations in visual cortex”^17^. The primary effects in these neuroimaging studies were found in higher level visual areas, not V1.

How has categorization-driven perceptual change been investigated empirically? Most previous research on categorization and perceptual change has employed artificial stimuli, such as simple, abstract forms, and tested for perceptual changes with only a small number of to-be-learned categories (most often two). Some studies have used more naturalistic examples, such as human faces. In such studies, the boundaries between categories have often been artificially constructed by the experimenter. One common approach is to generate stimulus sets by morphing between two or more ‘parent’ entities and defining membership by proximity along a continuum (e.g.,^3,12^). This method yields categories with systematically controlled levels of feature overlap, while also establishing clear rules for category membership.

Most categories encountered in everyday life, however, are complex and ill-defined. There may be no single rule or pattern that is universally distinguishing^37–39^. In multiple-category classification, properties that differentiate Category A from Category B may differ entirely from those that distinguish Category A from Categories C or D. The challenge of learning naturally occurring categories can be compounded by substantial within-category variation and by probabilistic relations between a given property and category membership. As a result, successful learning often demands extraction of complex relations and considerable information integration across stimulus properties. Natural domains also often involve large numbers of related categories, with complicated structures of similarity and difference.

Whether perceptual changes observed in constrained laboratory tasks also occur when people confront the complexity, variability, and ambiguity of real-world category structures remains an open question. Addressing this question requires testing perceptual learning in domains that are richly structured and practically significant. Here, we examined whether and how perception changes following multi-category classification training in a rich, naturally structured domain. We focused on the diagnosis of dermatological skin lesions, a domain that is both theoretically challenging and of considerable practical importance.

Understanding how perceptual expertise develops is especially important in medical image perception, where categorization among visually similar entities bears directly on patient outcomes (for reviews, see^40–42)^. In dermatology, classifying suspicious skin lesions involves integrating features such as symmetry, size, border irregularity, and color, yet these features often overlap across lesion types and vary within categories, complicating differential diagnosis, even for experts. While standard training incorporates explicit heuristics, such as the ABCD guidelines for melanoma detection which summarize key features for identification (A = Asymmetry, B = Border irregularity, C = Color variegation, D = Diameter generally greater than six millimeters^43)^, expertise in this and many real-world domains relies heavily on implicit perceptual learning^44–49^. Research on dermatological diagnosis in particular suggests low efficacy of explicit features and rules, and that classification expertise depends more on “non-analytical pattern recognition”^48^ rather than explicit reasoning.

In the present work, changes in the perception of skin lesion categories were measured by training medically naive observers on ten different skin lesion categories. Similarity ratings were collected from each participant before any category information was given and again after categorization training, allowing assessment of changes within and across categories. Because the diagnostic categories are rooted in naturally occurring biological phenomena, we might expect that items within the same category will exhibit greater baseline similarity than items from different categories. Consequently, perceptual change related to categorization would be reflected in increases in within-category similarity and decreases in between-category similarity.

### Category label considerations

Changes in perceptual experience resulting from learning have most often been assessed by measuring rated similarity for pairs of items before and after categorization training. One concern with this method is that similarity ratings might reflect altered task demands rather than true perceptual change. Goldstone, Lippa, and Shiffrin^3^ distinguished a *changed object description account*, where category learning truly alters object perception and representation, from a *strategic judgment bias account*, in which retrieving learned labels during similarity ratings biases judgments irrespective of appearance.

Investigators have used several methods to distinguish these accounts. Goldstone et al.^3^ measured changes in similarity ratings both among categorized items and in relation to a neutral, uncategorized item. They found evidence of changes in perceived similarity between categorized items and the uncategorized item as a result of training. Because participants had never been given a category label for the uncategorized item, they concluded that changes in similarity ratings indicated true perceptual change. More recently, Ashby et al.^1^ investigated the possible influence of both accounts by training participants on categories with either similarity-consistent (shared physical features within categories) or similarity-inconsistent (category membership orthogonal to features) structures. Changes in similarity judgments occurred in the similarity-consistent condition, but not in the inconsistent condition. The authors concluded that changes in similarity ratings primarily reflect “true changes in perception as attention is shifted toward category-relevant and away from category-irrelevant features to better align perceived similarity with category membership”^1^, p. 2] (cf.^24,25)^. Although there could be some strategic effects relating to category labels, the evidence suggests that changes in similarity ratings after perceptual training are primarily driven by true perceptual change.

We designed the present study to minimize and check for potential strategic judgment biases in several ways. Unlike most prior work, which has used small numbers of categories and instances, we used 10 categories and > 500 distinct images. Paired comparisons were used in categorization training, so two exemplars and two category names were presented in feedback on each trial. A given image appeared, on average, fewer than two times across 500 learning trials, making memorization of specific image-label pairs unlikely.

We also included a direct probe of labeling effects: a *label-overlap* manipulation in which several diagnostic categories shared overlapping terms in their names (e.g., *Basal Cell Carcinoma* and *Squamous Cell Carcinom*a; *Actinic Keratosis* and *Seborrheic Keratosis*). If learners relied heavily on labels during similarity judgments, label-overlap pairs should exhibit greater post-training similarity than non-overlap pairs (e.g., *Basal Cell Carcinoma* and *Wart*). Additionally, although some studies have used successive presentations of images in similarity rating trials, we presented images concurrently to keep all perceptual information simultaneously available during each similarity judgment. Finally, we related similarity ratings to the strength of learning across categories, as measured by participants’ post-training ability to classify wholly novel exemplars (not presented during training or in similarity ratings). We consider the relevance of this manipulation in the Discussion.

## Method

### Participants

Forty-nine undergraduate psychology students from the University of California, Los Angeles were recruited for participation in this study. After exclusions relating to attention checks and a required minimum in the learning phase of the experiment (see Exclusion criteria), there were 31 participants in the final sample. Participants provided informed consent and were awarded partial course credit for their participation. This study was determined to be exempt from full ethical review by the UCLA Office of the Human Research Protection Program and was conducted in accordance with the ethical standards of the Declaration of Helsinki.

### Materials

Stimuli consisted of dermoscopic images of skin lesions obtained from the MoleMap database (~ 2.5 M images, with image verification based on a combination of expert dermatologists, melanographers, and AI systems). A dermoscopic image is a magnified and illuminated image obtained by a dermoscope, which uses illumination from an angle to avoid reflections from the surface, with the result that deeper levels of the epidermis can be imaged.

**Fig. 1:**
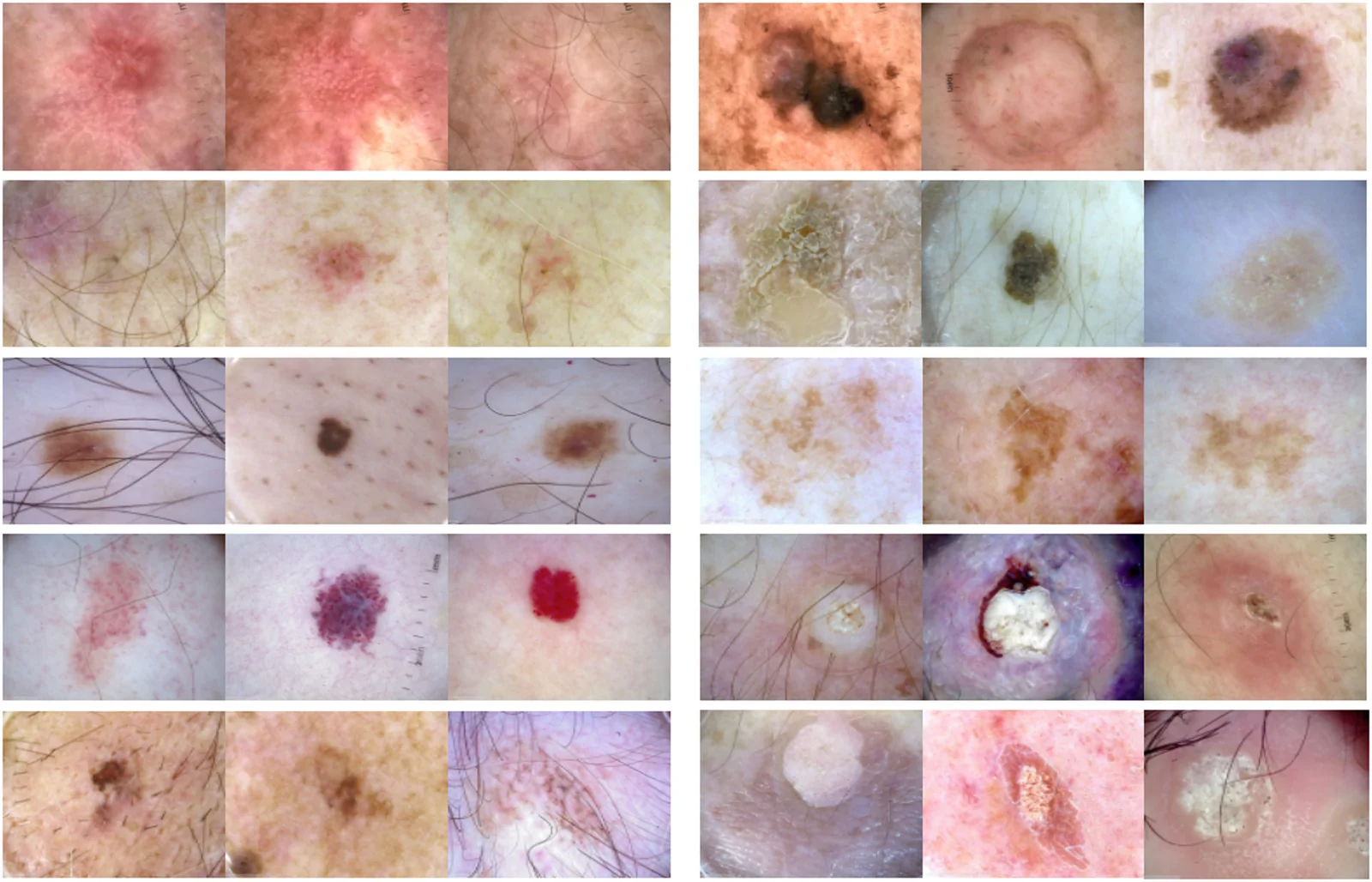
Skin lesion categories. Three examples from each of the ten categories are shown. In the first column, from top to bottom: Actinic Keratosis, Basal Cell Carcinoma, Benign Nevus, Haemangioma, Lentigo Maligna Melanoma. In the second column, from top to bottom: Nodular Melanoma, Seborrheic Keratosis, Solar Lentigo, Squamous Cell Carcinoma, Wart. All images were obtained from the MoleMap database.

Ten diagnostic categories of skin lesions were used: Actinic Keratosis, Basal Cell Carcinoma, Benign Nevus, Haemangioma, Lentigo Maligna Melanoma, Nodular Melanoma, Seborrheic Keratosis, Solar Lentigo, Squamous Cell Carcinoma, and Wart. Examples of each category can be seen in Fig. 1. The number of instances per category varied, with each category containing anywhere from 21 to 167 unique images, for a total of 715 available items. Images were chosen on the basis of original dermatologic diagnosis, verification via biopsy when appropriate, and good image quality. Variation in the number of instances per category was due to the numbers of instances fulfilling these criteria that were available in the database.

Of note, included within these categories were four category pairings that shared significant overlap in their names. These were: Actinic *Keratosis* and Seborrheic *Keratosis*, Basal *Cell Carcinoma* and Squamous *Cell Carcinoma*, Lentigo Maligna *Melanoma* and Nodular *Melanoma*, and *Lentigo* Maligna Melanoma and Solar *Lentigo*. We refer to these four pairings as *label overlap categories*. All other different-category pairings are *non-overlap categories*.

**Fig. 2:**
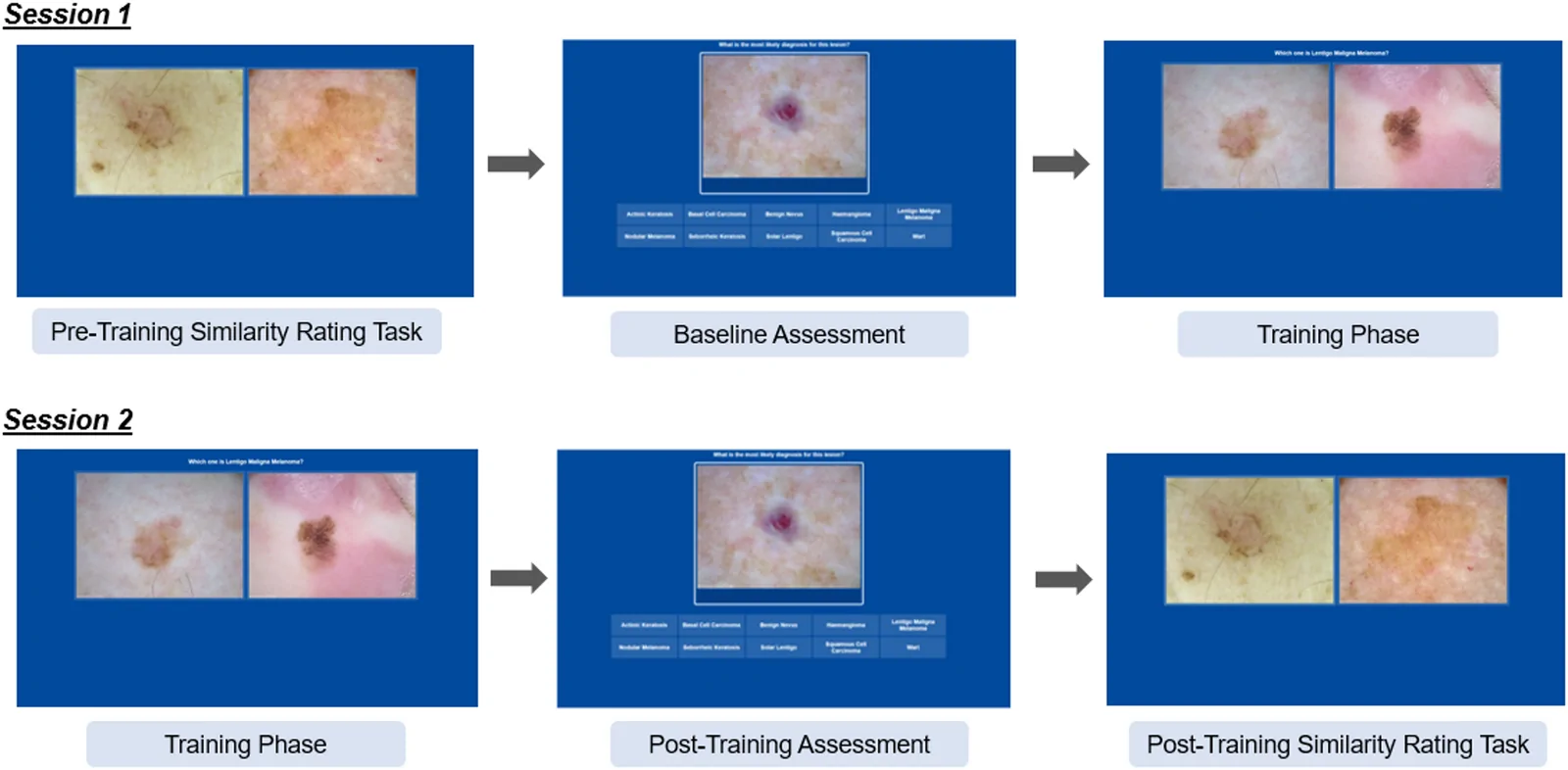
Procedure overview. Participants completed three different tasks–a similarity rating task, assessment of skin lesion classification, and classification training–twice each across two experimental sessions. Example trials for each task are shown. In the assessment task, the text at the top of the image reads: “What is the most likely diagnosis for this lesion?” In the training phase, the text on top of the image reads: “Which one is Lentigo Maligna Melanoma?”.

### Design & procedure

This study utilized a within-subjects design across two sessions completed two days apart. Figure 2 shows an overview of the procedure. Session 1 included a pre-training similarity rating task, a baseline assessment on classification of skin lesions, and the first half of categorization training. Session 2 included the second half of training, a post-training classification assessment, and a post-training similarity rating task. All participants completed this study online.

### Similarity rating task

Each trial began with a 500-ms blank screen, followed by side-by-side presentation of two images for 4,000 ms. Images were then removed, and participants rated similarity by clicking directly on a 1–9 scale labeled “Most Dissimilar” to “Most Similar.” Only integer responses were accepted. Six practice trials were given to provide participants with a feel for the task and provide some exposure to the range of stimuli that they would encounter. Practice trials were selected to include two low-similarity pairings, two intermediate-similarity pairings, and two high-similarity pairings. These image pairs were selected by the experimenter and independently rated by two research assistants familiar with the range of stimuli. Each category was shown at least once in practice. The main task comprised 168 trials: 30 same-category pairings (each category paired with itself 3 times), 135 different-category pairings (each category paired with each other category 3 times), and three attention-check trials with identical images. Trials were randomized, with a break at midpoint. The same image pairings were used at pre- and post-training. Participants were given no feedback or category information at any point in the task.

To capture real-world variability, images spanned a range of diagnostic difficulties while remaining reasonably identifiable. Selection was guided by prior image-level performance data ^50^, prioritizing images with documented mutual confusions (e.g., Wart vs. Nodular Melanoma) for different-category pairings, while excluding borderline cases that were too ambiguous for naive learners (mean accuracy < 10% throughout training). Based on availability and these criteria, the similarity task included 217 distinct images.

### Training phase

Following the pre-training similarity task, participants completed 500 learning trials split equally across two training sessions. On each learning trial, two skin lesion images, each from a separate category, were presented side by side under the prompt “Which one is [Category Name].” Participants were instructed to click on the image of the lesion they believed matched the one named in the prompt. Participants were given up to 30 s to select their answer. Immediate feedback was provided after each trial in the form of a green checkmark appearing alongside their answer choice when correct and a red ‘X’ appearing when incorrect. Additionally, the correct category label was presented under both presented images. Feedback was displayed for up to 10 s; however, participants could advance to the next trial at any point. This comparison-based learning approach was motivated by evidence that paired comparisons benefit perceptual learning in this domain^50^. Each category was shown 100 times in training, 50 times as the correct answer and 50 times as the incorrect, distractor option, with randomized category ordering and pairings.

### Classification assessments

Participants completed 20 single-item classification trials immediately before training (baseline) and after training (post-training). Each assessment used two images per diagnostic category. All 10 category labels were displayed below the presented image on each trial, and participants had up to 30 s to select a response. No feedback was provided. All assessment items were novel (i.e., previously unseen images).

### Exclusion criteria

Only participants who completed both sessions of the experiment were included in the analyses. Participants were excluded if they gave a rating below 8 out of 9 on any attention check during the similarity trials or if they showed less than a 30% improvement in skin lesion classification accuracy from baseline to the post-training assessment. In total, 15 participants were excluded for failing at least one attention check. Of the remaining participants, an additional 3 participants were removed for failing to meet the learning criterion. The final sample included 31 participants.

### Dependent measures and data analyses

For each participant, performance on the baseline and post-training assessments, measured as the percentage of items correctly classified, was recorded. For the similarity rating task, ratings were averaged across same-category pairings and different-category pairings for each participant at both phases of the experiment. Contrasts were planned for both pairing types at the two different similarity rating phases.

Assessment accuracy and similarity ratings for same-category pairings and different-category pairings were also calculated separately for each category. To examine whether the magnitude of similarity change corresponded to participants’ learning, a linear mixed-effects regression was conducted predicting composite similarity change from learning strength. Correlation analyses were also conducted to investigate possible relationships between similarity changes and assessment performance. Finally, differences in reported similarity were compared between label-overlap and non-overlap pairings. Condition differences were evaluated using standard parametric measures with two-tailed tests and alpha set at 0.05. Effect sizes, calculated as Cohen’s *d*, are reported for each difference.

## Results

### Training and assessment accuracy

Accuracy for paired comparison learning trials in the training phase increased from 67.90% (SD = 11.67) in the first 20-trial block to 89.35% (SD = 7.39) in the final block. Performance on the classification-based assessments increased significantly from a mean of 17.26% (*SD* = 10.87) prior to training to 72.74% (*SD* = 11.75) at the post-training assessment, *t*(30) = 22.58, *p* <.001, *d* = 4.90.

We note that performance on the baseline assessment was significantly above chance (10%) prior to training. Because these categories correspond to real-world entities, participants may have had varying degrees of prior exposure. To determine whether above-chance performance was driven by any particularly salient or familiar category, we examined category-level accuracy. A repeated-measures ANOVA showed no reliable differences across categories, *F*(9, 270) = 0.74, *p* =.677, indicating that no single category systematically contributed to elevated baseline accuracy.

In addition, baseline accuracy for each category was compared against chance performance (10%) using one-sample t-tests. Only one category (Lentigo Maligna Melanoma) was classified significantly above chance (*M* = 25.81%, *SD* = 33.84%; *t*(30) = 2.60, *p* =.014; not corrected for multiple comparisons). Because baseline accuracy for each category was based on only two trials per participant, an average accuracy of 25.81% indicates that the majority of participants did not correctly classify either Lentigo Maligna Melanoma exemplar. Above-chance performance was driven by a minority of participants (10 correctly classified one instance; 3 correctly classified both).

### Similarity ratings

**Fig. 3:**
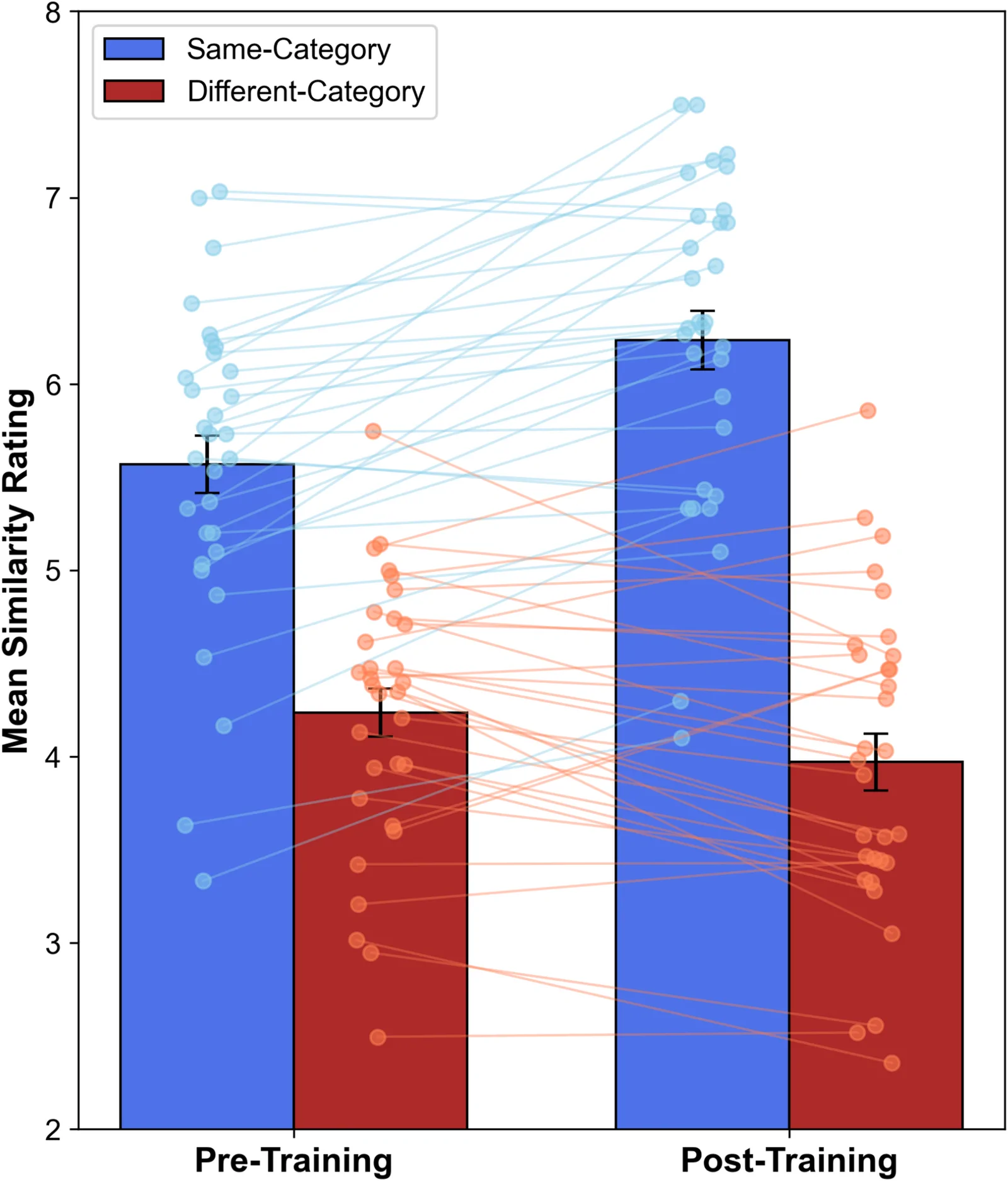
Mean similarity ratings for same- and different-category pairings before and after categorization training. Error bars show ± 1 SE. Individual subject data are plotted and connected from pre- to post-training.

Figure 3 shows mean similarity ratings for same-category and different-category pairs before and after categorization training. Even prior to training, same-category pairs were rated more similar than different-category pairs. Most relevant to our aims, same-category similarity increased and different-category similarity decreased following training.

To quantify the overall effect of training on perceived similarity, we computed a composite similarity change score: within-category change (post – pre) was combined with the sign-reversed between-category change (pre – post). This measure aggregates changes in perceived similarity in the predicted directions (i.e., increased within-category similarity and decreased between-category similarity). The composite similarity change score was M = 0.93 (SD = 0.61), significantly greater than zero (no change), *t*(30) = 8.55, *p* <.001, with a large effect size (*d* = 1.54).

To examine the effects separately for same- and different-category pairings, we conducted a 2 (pairing: same vs. different) X 2 (phase: pre vs. post) repeated-measures ANOVA on the similarity ratings. This analysis revealed main effects of pairing, *F*(1, 30) = 376.04, *p* <.001, η_p_^2^ = 0.93, indicating that, overall, same-category pairs were rated as more similar than different-category pairs. There was also a significant main effect of phase, *F*(1, 30) = 5.13, *p* =.031,η_p_^2^ = 0.15, indicating that across both pairing types, similarity ratings changed from pre- to post-training. Critically, there was a robust interaction of pairing type by phase, *F*(1, 30) = 73.08, *p* <.001, η_p_^2^ = 0.71, confirming that training differentially affected same- and different-category similarity ratings.

Pre-training, same-category pairings (*M* = 5.57, *SD* = 0.86) were rated higher than different-category pairings (*M* = 4.24, *SD* = 0.72), *t*(30) = 17.66, *p* <.001, *d* = 1.68. This pattern was amplified by training, with same-category pairings (*M* = 6.23, *SD* = 0.87) rated as significantly more similar than different-category pairings (*M* = 3.97, *SD* = 0.84), *t*(30) = 17.14, *p* <.001, *d* = 2.64. Within pairing types, same-category similarity increased from pre- to post-training by 0.67 points (*SD* = 0.60), *t*(30) = 6.22, *p* <.001, *d* = 0.76. Different-category similarity decreased by 0.26 points (*SD* = 0.56), *t*(30) = −2.62, *p* =.014, *d* = 0.35.

### Similarity ratings by category

Table 1 reports category-wise similarities pre- and post-training and corresponding changes. Within-category increases varied by category (Haemangioma: +2.21; Basal Cell Carcinoma: −0.37). This pattern was confirmed by a repeated-measures ANOVA, which revealed a significant main effect of category for same-category pairings, *F*(9, 270) = 11.11, *p* <.001. Variation for different-category pairings was less pronounced, as each measure averaged a category’s rating changes relative to the other nine. Benign Nevus showed the largest reduction (−0.58), whereas the smallest was Nodular Melanoma, which showed no change. A significant main effect of category was also observed for different-category pairings, *F*(9, 270) = 3.08, *p* =.002. The mean pre- and post-training similarity ratings, broken down by each pairwise different-category pairing, are provided in Table S1 in the Supplementary Materials. Category size (number of unique training exemplars available) did not predict change in similarity for same-category pairings, *r*(8) = − 0.38, *p* =.284, or different-category pairings, *r*(8) = − 0.39, *p* =.264.

**Table 1:** Similarity ratings by category. Mean similarity scores before and after training, along with the change in similarity (calculated as post-training score minus pre-training score), are presented for each category.

Category	Same category pairings			Different category pairings		
	Pre-training similarity	Post-training similarity	Similarity change	Pre-training similarity	Post-training similarity	Similarity change
Actinic Keratosis	7.05	7.81	+0.76	4.19	3.80	−0.39
Basal Cell Carcinoma	5.59	5.22	−0.37	4.23	3.95	−0.28
Benign Nevus	6.99	6.99	0.00	4.77	4.19	−0.58
Haemangioma	5.66	7.87	+2.21	4.13	3.94	−0.19
Lentigo Maligna Melanoma	5.25	5.22	−0.03	4.36	4.07	−0.29
Nodular Melanoma	5.95	7.68	+1.73	3.97	3.97	0.00
Seborrheic Keratosis	3.84	3.73	−0.11	3.93	3.87	−0.05
Solar Lentigo	6.73	6.46	−0.27	4.72	4.46	−0.26
Squamous Cell Carcinoma	4.74	5.95	+1.21	3.56	3.24	−0.32
Wart	3.89	5.44	+1.55	4.51	4.24	−0.27

**Table 2:** Similarity rating change for label-overlap and non-overlap category pairings. Similarity ratings are divided among the four label-overlap category pairings and the remaining 41 non-overlap labeled category pairings.

Category pairing	Pre-training similarity	Post-training similarity	Similarity change
Actinic Keratosis vs. Seborrheic Keratosis	4.35	4.57	+0.22
Basal Cell Carcinoma vs. Squamous Cell Carcinoma	3.76	3.13	-0.63
Lentigo Maligna Melanoma vs. Nodular Melanoma	5.58	5.32	-0.26
Lentigo Maligna Melanoma vs. Solar Lentigo	6.69	6.35	-0.34
All other different-category pairings	4.15	3.88	-0.27

### Relationship between categorization performance and similarity changes

If perceptual learning of diagnostic categories drives upweighting and downweighting of stimulus properties to achieve accurate classification, these encoding changes should lead to changes in perceptual experience, such as perceived similarity. To examine whether the magnitude of similarity change corresponded to the degree of learning within participants, we fit a linear mixed-effects regression model predicting composite similarity change from learning strength at the participant by category level, including the number of unique examples seen for each category (category size) as a covariate. We performed the regression separately for both posttest accuracy and for accuracy during the learning phase. (We did so because posttest accuracy, with only two exemplars per category, could yield accuracies of only 0, 0.5 or 1 per category, possibly reducing the visibility of a linear relationship). The models included random intercepts for participants and categories to account for baseline differences across these factors.

Results indicated that accuracy during the learning phase significantly predicted similarity change (*b* = 3.25, *t*(126.7) = 3.00, *p* =.003), and posttest accuracy also predicted similarity change (*b* = 0.66, *t*(277.77) = 2.09, *p* =.034). Both results indicate that categories learned more successfully exhibited greater perceptual change in the expected direction. Category size did not significantly predict similarity change (*b* = −0.02, *t*(7.8) = −1.95, *p* =.087 in the model including learning-phase accuracy; *b* = −0.02, *t*(8.0) = −1.94, *p* =.089 in the model including posttest accuracy), suggesting that the effect of learning strength is not driven by differences in the number of exemplars per category.

We also sought to characterize further the relationship between similarity change and classification performance at the category level, where we asked whether different forms of similarity change might be differentially related to distinct components of categorization judgments for novel examples. To address this question, we applied concepts from signal detection theory (SDT), characterizing post-training classification performance in terms of hit rates and false alarm rates calculated separately for each category. Correctly classified exemplars were classified as hits, and trials with misclassified items were recorded as false alarms for the category whose label was incorrectly selected. Although the comparison is not exact due to differences in paradigms and number of categories (two vs. many), there is at least a conceptual analogy between learning effects that involve increasing hits vs. reducing false alarms in multi-category classification and efforts to distinguish signal enhancement from noise reduction in perceptual learning (e.g.,^51,52)^. In multi-category classification, if learning enhances within-category similarity, it may be reflected in classification of novel stimuli with greater hit rates, whereas enhancement of between-category distinctiveness may be reflected in lower false alarm rates.

Although within-category compression and between-category expansion often co-occur, in multidimensional representational spaces containing many categories they need not emerge uniformly across all category boundaries. Crucially, within-category compression does not imply uniform expansion from all neighboring categories. Geometrically, compression reduces within-category variance, whereas false alarm rates depend primarily on the distance to the nearest competing categories. In spaces with many categories, expansion can be selective (occurring for some category pairs but not others) allowing compression to increase hit rates even when false alarm rates remain high due to persistent overlap with specific neighbors.

**Fig. 4:**
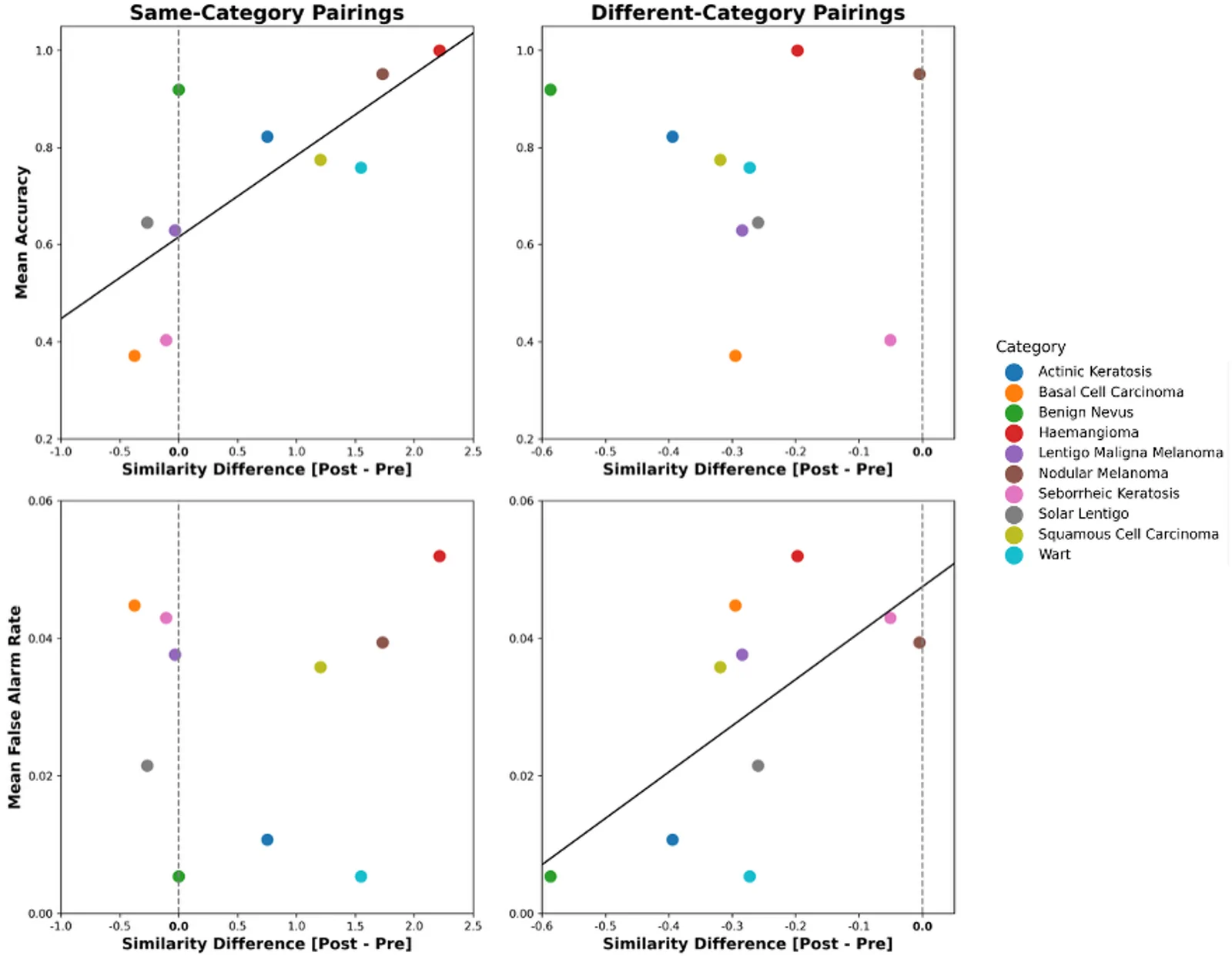
Posttest performance by similarity difference. *Top Row*: Mean accuracy in classifying novel exemplars on the post-training assessment is plotted against the mean similarity difference (post-training similarity minus pre-training similarity) for each category, separated into Same-Category Pairings and Different-Category Pairings. *Bottom Row*: Mean false alarm rate on the post-training assessment is plotted by similarity difference for each category. The solid black line represents the line of best fit for significant correlations, while the dotted grey line marks the expected position of categories if no change in similarity had occurred.

Figure 4 displays these relationships. For same-category pairings, classification accuracy correlated highly with similarity rating change, *r*(8) = 0.74, *p* =.015. Categories with the highest compression tended to be those with highest accuracy at the post-training assessment. No reliable correlation emerged between accuracy and expansion, *r*(8) = −0.18, *p* =.621. The latter result likely reflects the greater compression effect we found and its dominance in the overall accuracy data. Improvement in distinguishing differences did occur, but with 10 categories as response options, learning to encode key diagnostic features within each category may have been more important for accuracy.

We examined false alarm rates as a potentially more sensitive analysis for improvement in discerning differences. Greater between-category expansion predicted lower false-alarm rates, *r*(8) = 0.64, *p* =.047. No significant correlation was found between false alarms and compression, *r*(8) = 0.12, *p* =.747.

### Label overlap

Table 2 shows mean similarity change for each of the four label-overlap category pairings relative to all other different-category pairings. Similarity rating scores were averaged across all label-overlap and non-overlap different-category pairs for each participant. Mean similarity change was nearly identical for label-overlap (*M *=-0.25, *SD *= 0.79) and non-overlap category pairings (*M *= -0.27,* SD *= 0.56). A 2 (phase: pre- vs. post) X 2 (pair type: label-overlap vs. non-overlap) repeated-measures ANOVA showed a significant main effect of Phase, *F*(1, 30) = 5.60, *p* =.025 (overall decrease in different-category similarity), and a significant main effect of pair type, *F*(1, 30) = 92.72, *p* <.001 (label-overlap pairs were rated as more similar at both time points). Crucially, there was no phase by type interaction, *F*(1, 30) = 0.015, *p* =.903, indicating that training-induced similarity changes did not depend on label overlap.

## Discussion

We asked whether categorization training in a large, naturally structured domain yields changes in perceived appearance comparable to those observed with simplified laboratory stimuli. It does. Similarity ratings increased for items from the same category and decreased for items from different categories, providing evidence of both within-category compression and between-category expansion. Perceptual change was accompanied by strong improvements in classification performance, from 17% at baseline to 73% post-training on novel images.

Compression and expansion effect magnitudes varied by category. Differences likely reflect intrinsic variability within categories and the perceptual salience of diagnostic features prior to training. Using real medical images introduces meaningful heterogeneity: some categories are inherently more variable or more overlapping than others, which in turn affects both how easily they are learned and how much perceptual change is required to support accurate diagnosis. Categories that are already relatively consistent and distinct may require minimal perceptual reweighting, whereas more variable or confusable categories may require larger shifts in representational structure. In this sense, perceptual learning is expected to produce non-uniform changes, with restructuring emerging primarily where it is beneficial for improving task performance, rather than uniformly across all categories. Therefore, differences in perceptual change are not simply the result of whether a category is initially easy or difficult, but also of how much change is needed and what diagnostic information can be leveraged through learning.

Overall, compression effects were stronger but more variable across categories than expansion effects. The training format—trial-by-trial between-category comparison—may have preferentially directed attention to distinguishing features, placing less emphasis on discovering commonalities across same-category exemplars (cf.^53,54)^. Conversely, expansion effects appeared comparatively modest, but more consistent across categories. This may reflect our method of aggregating similarity ratings. To assess how a category was perceived relative to others, we averaged similarity ratings across all pairings involving that category. While this provided an overall measure of distinctiveness, it may have masked important variation among specific category pairings.

When examined at the pairwise level, between-category similarity changes showed substantial variability (e.g., Benign Nevus vs. Squamous Cell Carcinoma: −1.13; Nodular Melanoma vs. Squamous Cell Carcinoma: +0.98). Importantly, these effects arise within a high-dimensional representational space in which changes to one set of category relationships may be accompanied by changes in others. As a result, increased similarity for some pairs could reflect poorer learning of one or both categories involved; however, it may also reflect redistribution of representational distance as the system adapts to improve separation where it is most diagnostically useful.

For example, perceived similarity between Haemangioma and Nodular Melanoma increased by 0.87 points after training. In isolation, this pattern could be consistent with reduced discriminability between these categories. However, these same categories also showed the largest and third-largest composite similarity changes overall and achieved the highest and second-highest posttest classification accuracies. Taken together, this suggests that the increase in similarity did not reflect poor learning but occurred alongside strong evidence of successful learning elsewhere. More broadly, this pattern is consistent with non-uniform restructuring across the category space, in which improvements in distinguishing particularly confusable category pairs may be more critical for performance than achieving uniform separation across all categories.

### Perceptual change vs. label-driven judgment

Definitively separating perceptual from decisional contributions is challenging. While the purpose of this work is not to focus on a detailed discussion of similarity ratings as an index of perceptual change (see Goldstone et al.^3^ and Ashby et al.^1^ for extensive treatment of these issues), no single measure alone can definitively rule out either account entirely. Accordingly, we draw on multiple converging sources of evidence to support the interpretation that categorization-driven perceptual changes occurred in the present study.

Several features of our design argue against changes in similarity judgments reflecting strategic label retrieval rather than perceptual change. Participants were exposed to large numbers of categories and images with very sparse repetition, making memorization of specific exemplars unlikely. Additionally, we used naturalistic categories that required a degree of information integration to learn, rather than more artificial, rule-based categories in which deterministic rules could be learned and verbalized for all categories.

The similarity rating task itself did not present any category labels or provide feedback of any kind, placing no emphasis on explicit categorization decisions. Instead, it prompted participants to make judgments based on the perceptual appearance of the stimuli at the time of viewing, with no clear incentive to rely on or retrieve labels for the presented items. In addition, images were presented simultaneously during similarity ratings, which minimized the risk of sequential comparisons that could encourage reliance on recalled labels.

Crucially, our manipulation of overlapping category labels provided a direct test of label-based accounts. If shared labels had biased judgments, categories with related names should have shown increased similarity. Instead, between-category similarity decreased comparably for overlapping (–0.25) and non-overlapping (–0.27) labels, providing strong evidence that perceptual learning, rather than strategic labeling, drove the observed changes.

While the label-overlap analysis was intended to test whether participants relied on the experimenter-provided labels when making similarity judgments, one possible limitation of this manipulation would be if participants systematically ignored the shared parts of labels or invented their own internal labels for each category over the course of the learning phase. We have no evidence that this occurred, but it is imaginable. Importantly, however, the results of this analysis were corroborated by other participant- and category-level effects.

Categories that participants learned more successfully showed greater shifts in perceived similarity, suggesting that changes in perceptual representation reflected the strength of learning. This is intuitive: to perceive items within the same category as more similar, participants must have detected shared features, whereas perceiving categories as more distinct requires identifying distinctive features. These patterns persisted even when controlling for differences in the number of exemplars per category, demonstrating that the effects were not driven by category size, as might be expected if participants were relying on memorization of individual images and labels.

This interpretation is further corroborated by category-level effects. Compression and expansion varied systematically by category despite identical training exposure, with no relationship to category size. Moreover, changes in similarity correlated strongly with classification accuracy for novel, unlabeled images in the posttest. Because these stimuli were entirely new and never paired with labels, their alignment with similarity shifts indicates that the observed effects reflect genuine changes in perceptual encoding rather than criterion shifts or strategic label retrieval.

### Category learning and perceptual learning: cause and effect

The results of this study support the hypothesis that category learning leads to changes in perceptual appearance. How might this work? It is often asserted that cognition influences perception because knowledge, categories, past associations, etc. in the mind actually alter how incoming stimulation is experienced and represented. A standard illustration is the *memory color effect* (MCE): if a gray rectangular patch appears neutral in color, but a gray apple-shaped patch looks slightly reddish, one might infer that the concept *apple* (and its typical redness) has altered the percept. Our aim here is not to affirm or deny the MCE, whose existence remains a matter of debate (see^55–57)^. The point is that if an MCE occurs, it would be a clear case of non-perceptual cognitive contents distorting or adding to incoming information to be more consistent with existing categories. Following Pylyshyn^58^, we use *cognitive penetration* to denote the claim that cognitive states (e.g., beliefs, goals, category labels) can alter perceptual appearance; for a recent critical review, see^59^.

We believe that our results are better understood on a different account—a *structure-driven perceptual learning account*—according to which perceptual change is driven primarily by categories in the world and task structure, not by internal category labels or beliefs. Mechanistically, this tuning can be understood as selective reweighting and differentiation of information^8,9,20,21,60^. This explanation has much in common with deep convolutional neural networks (deep learning systems), that have achieved unprecedented success in object categorization in artificial intelligence (for a review, see^22)^, including medical image classification in general^61^ and dermatological diagnosis in particular^62^. In short, perceptual change follows from interacting with external category structure; environmental consequences including category labels serve as feedback or training signals rather than as the proximate cause of altered appearance.

Variants of this account, which have been called *differentiation*^60^ or *selective reweighting* (e.g.,^63^), have two key features that differ from a cognitive penetration account. One is that categories do drive changes in perception, but these are not categories “in the head” or mental contents; they are categories in the world and/or in the structure of a task. As a result of the demands of and feedback from classification tasks in the world, corresponding mental categories may form or be modified. Second, this account involves no distortion or falsification of the input. Accurate categorization of dermatological lesions, for example, benefits not from distorted input, but from progressive differentiation, selection, and automatic pickup of diagnostic information. Perceptual learning in this context reflects improved *sensitivity* in the signal detection sense, whereas distorting input to fit pre-existing concepts or experiences reflects a shift in *criterion* or *bias*. Perceptual appearance changes because the discovery, selection, and reweighting of diagnostic information changes perceptual descriptions. In category learning, the causal arrow typically runs from perception to category – experience tunes information pickup to environmental and task structures, selectively altering perceptual encoding and yielding new or sharpened categories.

A structure-driven perceptual learning account provides a better explanation than a cognitive penetration account for the current results and for many reported influences of categorization on perception. We do not take a position about whether this account applies to all such claims. But it is important to realize that an influence of categories on perception does not imply a cognitive penetration view. In tasks like ours, and many others involving interactions of perception and categorization, the relevant categories are not “in the head” but in the *world*. Learning progresses by making better use of information, not the reshaping of perception to match some pre-existing mental content. In this view of perceptual learning, learning leads perception (and categorization) to be more in correspondence with the physical input^60^. The results reported here indicate that the improvement in the perceptual extraction of information leads not only to better performance but to changes in the way the objects in a domain are perceptually experienced.

## Supplementary Information

Below is the link to the electronic supplementary material.

Supplementary Material 1

## Data Availability

Data for this study have been made available on the Open Science Framework and can be accessed at: https://osf.io/wpt47
