# Supplementary material for: Categorization-driven perceptual learning changes perceived similarity in the naturalistic domain of skin lesion diagnosis
Source: Sci Rep. 2026 Jul 31;16:35405. doi: 10.1038/s41598-026-59055-z (PMC13719495; doi:10.1038/s41598-026-59055-z)
Supplement: Supplementary file 1 [file 41598_2026_59055_MOESM1_ESM.pdf]

Category Pairing			*Pre-Training*	*Post-Training*	*Difference*
Actinic Keratosis	vs.	Basal Cell Carcinoma	4.99	4.94	-0.05
Actinic Keratosis	vs.	Benign Nevus	3.83	2.99	-0.84
Actinic Keratosis	vs.	Haemangioma	2.94	2.95	0.01
Actinic Keratosis	vs.	Lentigo Maligna Melanoma	4.30	3.57	-0.73
Actinic Keratosis	vs.	Nodular Melanoma	2.34	1.81	-0.54
Actinic Keratosis	vs.	Seborrheic Keratosis	4.35	4.57	0.22
Actinic Keratosis	vs.	Solar Lentigo	5.43	5.34	-0.09
Actinic Keratosis	vs.	Squamous Cell Carcinoma	4.56	3.65	-0.91
Actinic Keratosis	vs.	Wart	5.01	4.40	-0.61
Basal Cell Carcinoma	vs.	Benign Nevus	4.22	3.73	-0.48
Basal Cell Carcinoma	vs.	Haemangioma	6.04	6.01	-0.03
Basal Cell Carcinoma	vs.	Lentigo Maligna Melanoma	3.47	3.39	-0.09
Basal Cell Carcinoma	vs.	Nodular Melanoma	2.76	2.67	-0.10
Basal Cell Carcinoma	vs.	Seborrheic Keratosis	4.04	3.60	-0.44
Basal Cell Carcinoma	vs.	Solar Lentigo	4.45	4.47	0.02
Basal Cell Carcinoma	vs.	Squamous Cell Carcinoma	3.76	3.13	-0.63
Basal Cell Carcinoma	vs.	Wart	4.34	3.60	-0.74
Benign Nevus	vs.	Haemangioma	5.26	4.60	-0.66
Benign Nevus	vs.	Lentigo Maligna Melanoma	5.13	4.74	-0.39
Benign Nevus	vs.	Nodular Melanoma	6.17	5.15	-1.02
Benign Nevus	vs.	Seborrheic Keratosis	4.31	3.59	-0.72
Benign Nevus	vs.	Solar Lentigo	5.83	6.00	0.17
Benign Nevus	vs.	Squamous Cell Carcinoma	3.30	2.17	-1.13
Benign Nevus	vs.	Wart	4.92	4.71	-0.22
Haemangioma	vs.	Lentigo Maligna Melanoma	3.37	3.15	-0.22
Haemangioma	vs.	Nodular Melanoma	4.99	5.86	0.87
Haemangioma	vs.	Seborrheic Keratosis	2.04	1.97	-0.08
Haemangioma	vs.	Solar Lentigo	3.65	3.44	-0.20
Haemangioma	vs.	Squamous Cell Carcinoma	2.95	2.39	-0.56
Haemangioma	vs.	Wart	5.97	5.05	-0.91
Lentigo Maligna Melanoma	vs.	Nodular Melanoma	5.58	5.32	-0.26
Lentigo Maligna Melanoma	vs.	Seborrheic Keratosis	5.96	5.80	-0.16
Lentigo Maligna Melanoma	vs.	Solar Lentigo	6.69	6.35	-0.33
Lentigo Maligna Melanoma	vs.	Squamous Cell Carcinoma	2.32	2.18	-0.14
Lentigo Maligna Melanoma	vs.	Wart	2.41	2.16	-0.25
Nodular Melanoma	vs.	Seborrheic Keratosis	2.92	2.73	-0.19
Nodular Melanoma	vs.	Solar Lentigo	3.19	2.67	-0.53
Nodular Melanoma	vs.	Squamous Cell Carcinoma	4.72	5.70	0.98
Nodular Melanoma	vs.	Wart	3.10	3.84	0.74
Seborrheic Keratosis	vs.	Solar Lentigo	5.15	5.27	0.12
Seborrheic Keratosis	vs.	Squamous Cell Carcinoma	1.76	2.17	0.41
Seborrheic Keratosis	vs.	Wart	4.38	4.71	0.33
Solar Lentigo	vs.	Squamous Cell Carcinoma	3.02	2.22	-0.81
Solar Lentigo	vs.	Wart	4.81	4.09	-0.72
Squamous Cell Carcinoma	vs.	Wart	5.65	5.57	-0.08

Table S1. Similarity Ratings for all Different-Category Pairings. Mean similarity scores before and after training, along with the change in similarity (calculated as post-training score minus pre-training score), are presented for each different-category pairing.
